# Coagulation Pathways in Neurological Diseases: Multiple Sclerosis

**DOI:** 10.3389/fneur.2019.00409

**Published:** 2019-04-24

**Authors:** Nicole Ziliotto, Francesco Bernardi, Dejan Jakimovski, Robert Zivadinov

**Affiliations:** ^1^Department of Life Sciences and Biotechnology, University of Ferrara, Ferrara, Italy; ^2^Department of Neurology, Jacobs School of Medicine and Biomedical Sciences, Buffalo Neuroimaging Analysis Center, University at Buffalo, State University of New York, Buffalo, NY, United States; ^3^Clinical Translational Science Institute, Center for Biomedical Imaging, University at Buffalo, State University of New York, Buffalo, NY, United States

**Keywords:** multiple sclerosis, coagulation, extrinsic pathway, intrinsic pathway, coagulation inhibitors, fibrinolytic pathway

## Abstract

Significant progress has been made in understanding the complex interactions between the coagulation system and inflammation and autoimmunity. Increased blood-brain-barrier (BBB) permeability, a key event in the pathophysiology of multiple sclerosis (MS), leads to the irruption into the central nervous system of blood components that include virtually all coagulation/hemostasis factors. Besides their cytotoxic deposition and role as a possible trigger of the coagulation cascade, hemostasis components cause inflammatory response and immune activation, sustaining neurodegenerative events in MS. Early studies showing the contribution of altered hemostasis in the complex pathophysiology of MS have been strengthened by recent studies using methodologies that permitted deeper investigation. Fibrin(ogen), an abundant protein in plasma, has been identified as a key contributor to neuroinflammation. Perturbed fibrinolysis was found to be a hallmark of progressive MS with abundant cortical fibrin(ogen) deposition. The immune-modulatory function of the intrinsic coagulation pathway still remains to be elucidated in MS. New molecular details in key hemostasis components participating in MS pathophysiology, and particularly involved in inflammatory and immune responses, could favor the development of novel therapeutic targets to ameliorate the evolution of MS. This review article introduces essential information on coagulation factors, inhibitors, and the fibrinolytic pathway, and highlights key aspects of their involvement in the immune system and inflammatory response. It discusses how hemostasis components are (dys)regulated in MS, and summarizes histopathological post-mortem human brain evidence, as well as cerebrospinal fluid, plasma, and serum studies of hemostasis and fibrinolytic pathways in MS. Studies of disease-modifying treatments as potential modifiers of coagulation factor levels, and case reports of autoimmunity affecting hemostasis in MS are also discussed.

## Introduction

The complex physiological process of hemostasis involves several pathways in which procoagulant and anticoagulant forces are maintained in a constant equilibrium by autoregulation. In fact, hemostasis allows the vascular wall to provide anticoagulant blood containment until damage causes significant activation of coagulation, the confined formation of blood clot with hemorrhage cessation, and removal of that clot after the restoration of vascular integrity (1).

**Figure 1:**
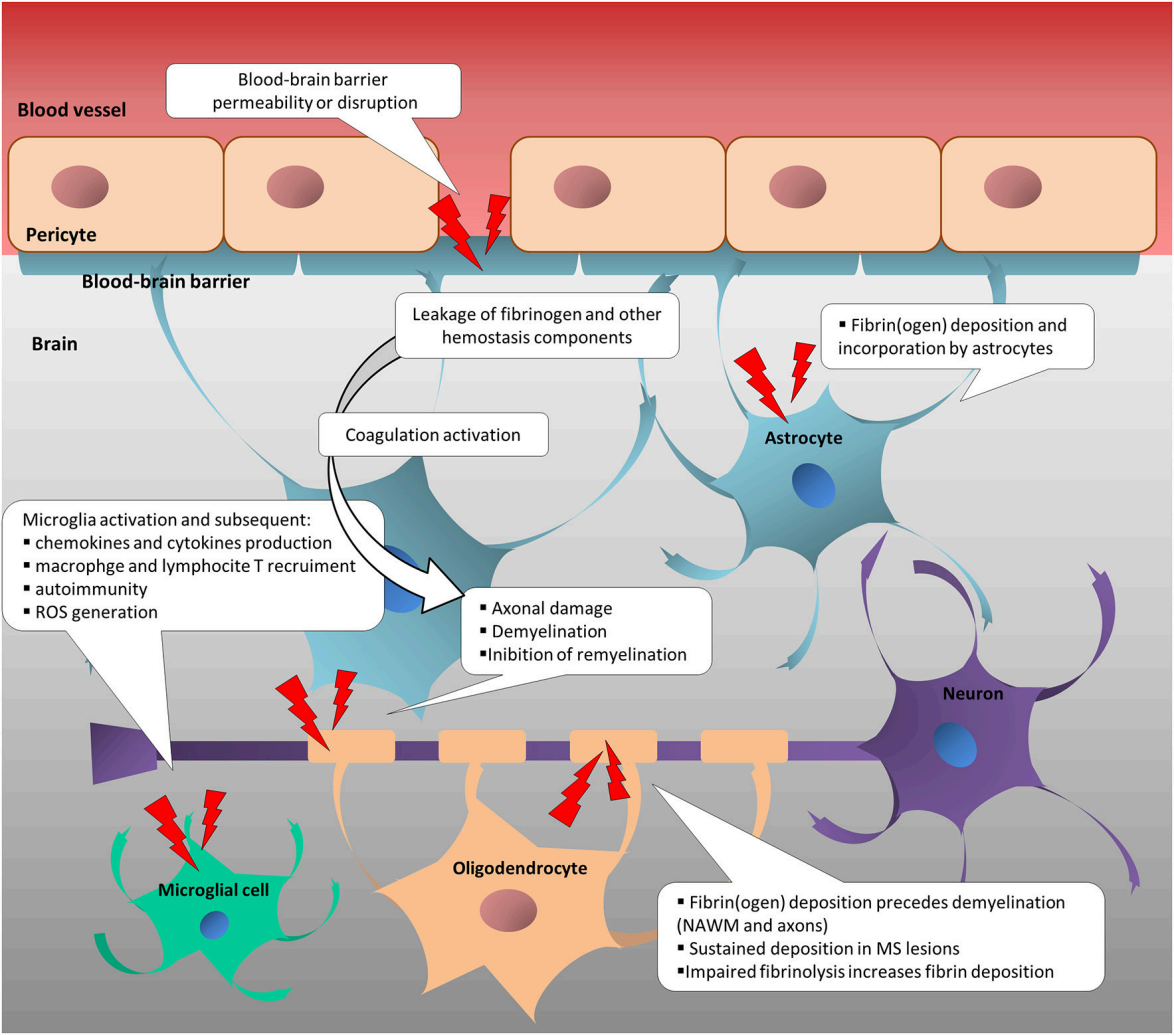
Change in neurovascular interface is involved in inflammatory, immune and neurodegenerative responses in multiple sclerosis. Disruption or increased permeability of blood-brain barrier cause the leakage of hemostasis components into the brain parenchyma, which triggers the coagulation cascade. In turn, hemostasis components foster the inflammatory response and the immune activation, sustaining neurodegenerative events in MS. NAWM, normal-appearing white matter; ROS, reactive oxygen species.

Increased blood-brain-barrier (BBB) permeability is a feature of several neurological diseases, and one of the first events that characterizes multiple sclerosis (MS) pathogenesis (2–5), leading to the irruption of coagulation/hemostasis factors into the central nervous system (CNS) (6). This, in turn, potentially triggers leakage of hemostasis components into the brain parenchyma, which potentially triggers the coagulation cascade. Besides their cytotoxic deposition, hemostasis components cause an inflammatory response and immune activation, sustaining neurodegenerative processes in MS (Figure 1) (6–12). Coagulation and inflammation are characterized by multiple links, and coagulation proteins and their fragments may promote neurodegeneration (12, 13). Preclinical models provide (albeit with some limitations) an informative means to investigate the pathophysiology of human diseases, and those mimicking MS have received attention in the last 3 decades. In particular, an increasing number of studies, largely based on animal models (14), have provided insights into the tight relationship among vasculature alterations, neuroinflammation, neuroimmunology, and neurodegeneration. Nevertheless, they only partially contribute to the relation between hemostasis components and experimental evidence in MS patients. This review article focuses on coagulation pathways in MS patients and related animal models. Current knowledge of how coagulation factors, coagulation inhibitors, and components of the fibrinolytic pathway are (dys)regulated in MS patients is reviewed and missing or inconsistent information is highlighted to guide future research.

## Coagulation Cascade Essentials

Before exploring the contribution of coagulation components in the pathophysiology of MS, it is important to consider the basic physiology of coagulation. Coagulation occurs as a complex network of overlapping reactions tightly localized on specific cell surfaces. It is often still represented as a one-way Y-shaped model as proposed in the 1960s (15, 16). Although an oversimplification, this model posits two distinct pathways, so-called “extrinsic” and “intrinsic,” that converged into a “common” one. Here, the interactions of inactive procoagulant mediators enable a sequential cascade of proteolytic events leading to their activation and the final fibrin and blood clot formation. The extrinsic pathway was so named because it requires an external factor (from the extravascular tissue), while the intrinsic pathway includes factors that are already present in the blood. In contrast to this commonly cited model, in the actual *in-vivo* process, extrinsic, and intrinsic pathways do not work independently and the pro-coagulant mediators, once activated, support the exponential amplification and propagation of the system with several interactions and feedback loops (17, 18). Although the activity in plasma of pro-coagulant factors of extrinsic and intrinsic pathways can be measured separately using clinically available coagulation tests such as partial thromboplastin time (PT) and activated partial thromboplastin time (aPTT), respectively (19), these laboratory tests do not accurately reflect the *in-vivo* situation (17). In fact, they force the system into a controlled condition on platelet-poor plasma through the exogenous supply of reagents (tissue factor/thromboplastin, phospholipids, calcium, and micronized silica) to assess the activity level of a certain factor.

In order to form a blood clot *in-vivo*, platelets and coagulation factors must communicate and support each other (20). Tissue factor (TF), the main trigger of the process responsible for the initial acceleration of cascade activation, is kept hidden on subendothelial cells until vascular damage exposes it (18). Once exposed, it promotes the activation of platelets and their recruitment into the clot (21). In turn, platelets mediate pro-coagulant functions through the release of additional coagulation factors and by the release of negatively charged phospholipids that are required cofactors for the proteolytic reactions of coagulation factors (22).

The procoagulant mediators that initiate, amplify, and propagate this cascade exist as proenzymes (also known as zymogens) in the blood (22). Under normal conditions, a basal activation of coagulation factors takes place, but it leads to an “idling” coagulation (18), which does not escalate to full clot formation. This occurs because the biochemical reactions are several orders of magnitude less efficient without the procoagulant mediators.

In summary, (1) fibrin can be produced only as a result of the complex interplay of coagulation factors, and (2) to productively trigger coagulation, cell surface exposure is necessary (TF-bearing membranes and platelets).

Based on the above, one of the main questions relevant to MS is how the coagulation cascade is triggered in the CNS.

### Extrinsic Coagulation Activation and Implication for Damage Within the CNS

The initiation of the “extrinsic” coagulation pathway requires “extravascular” TF, also known as Factor (F)III, thromboplastin, or CD142. It is important to note that the names of coagulation factors, identified with roman numerals, reflect the order in which they were discovered and not the biological order of the “sequential cascade” of proteolytic events. TF is highly expressed on the surfaces of medial and adventitial cells, acting as the trigger for arresting bleeding under damaging circumstances (1).

Surprisingly, low levels of TF in an inactive configuration (cryptic state) may be found on endothelial cells and blood cells including platelets, lymphocytes, monocytes, macrophages, granulocytes, and neutrophils (23–25). Additionally, TF has been found circulating in TF-bearing microparticles that are released from cells or as a soluble protein generated by alternative splicing of TF mRNA (26). Overall induction of soluble TF is stimulated during sepsis in response to bacteria, or during various chemokine- and cytokine-induced inflammatory states (27).

It has been suggested that decryption, which leads to the procoagulant activity of circulating TF, may depend on different mechanisms including a change in phospholipid environment, TF oxidation/reduction modifications, and TF dimerization (23, 28–30). Circulating microparticles may contribute to the formation of micro-thrombi (31). This has been suggested as one of the physiological defense strategies against bacteria, promoting so-called immunothrombosis in which the coagulation traps the pathogens, thereby preventing its spreading, and supporting the immune response (32). Uncontrolled activation of immunothrombosis, related to sepsis, cancer, or inflammatory states causes pathological conditions with undesired intravascular clotting contributing to pro-thrombotic risk (33).

**Figure 2:**
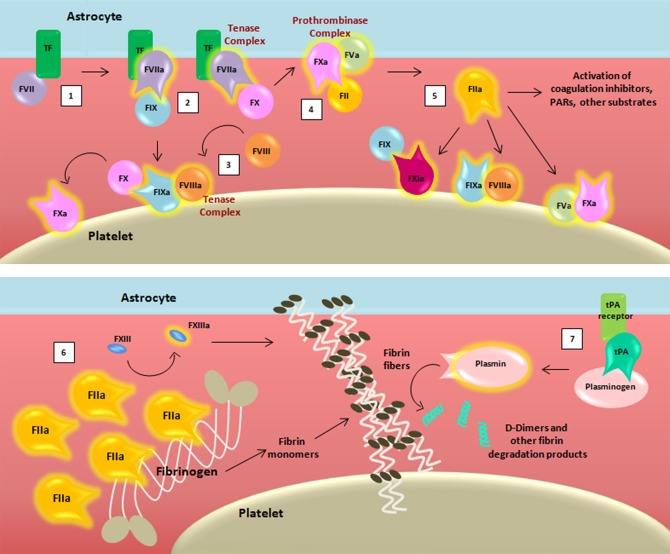
Schematic representation of the coagulation cascade and fibrinolytic pathway after blood-brain barrier damage. The coagulation cascade is activated (1) when the TF binds to its ligand, (activated) factor (F)VII, thus forming, together with membranes, a mature active binary complex (TF:FVIIa). The TF:FVIIa complex allows to cleave and activate on one side FIX and on another FX (2). TF:FVIIa:FXa is able to activate the cofactor FVIII (3) which forms a complex with the FIXa (FIXa:FVIIIa) providing a feedback loop for FX activation. The assembly FXa:FVa, converts prothrombin (FII) into thrombin (FIIa) (4). The initial amount of thrombin exerts its proteolytic action on FXI, FV, FVIII, and other substrates (5). Then the massive thrombin generation reaches a sufficient concentration to convert fibrinogen (FI) into fibrin monomers (6). The organized three-dimensional assembly of monomers in protofibrils and fibrin fibers produces the blood clot. Cross-linking stabilization of fibrin clot requires FXIII activated (FXIIIa) by thrombin activity. Coagulation complexes depend on lipids, exemplified by the platelet membrane. The dissolution of the fibrin fibers is mediated by the fibrinolytic system (7). Tissue-type plasminogen activator (tPA) converts plasminogen into plasmin which cleaves fibrin to soluble degradation products among those the D-dimers.

Given these observations, the tight relation between coagulation, inflammation, and immunity can already be appreciated in the vascular compartment alone. However, prominent expression of TF is also known to occur in the human brain (34, 35), and studies in mice have demonstrated that astrocytes are the primary cellular source of TF, suggesting their role in cerebral hemostasis (36). The breakdown of BBB that characterizes the MS disease process exposes the TF of astrocytes, which can promote activation of the coagulation cascade. The cascade in turn requires activated membranes to support biochemical reactions, canonically provided by platelets (Figure 2) (21).

For the sake of simplicity, we have chosen to omit reporting some of the intermediate cleaved forms of clotting factors, as well as their isoforms. Transmembrane TF of astrocytes binds its ligand FVII, activating and allosterically modifying it to form a mature active binary complex (TF:FVIIa). The TF:FVIIa complex cleaves and activates from one side FIX and from another FX, both present in the blood as zymogens (37). TF-FVIIa-nascent FXa complex activates FVIII (38). FVIIIa forms a complex with FIXa (FIXa:FVIIIa) providing a feedback loop for FX activation. Because of the ability to activate FX, both TF:FVIIa and FIXa:FVIIIa are called tenase complexes.

On the surface of TF-cells, FXa is released from TF:FVIIa and it associates with its cofactor FVa leading to the assembly of the FXa:FVa complex. The initial trace of FVa may derive from partially activated platelets (39), by proteases that are not involved in coagulation (40), or by FXa (41). The “common” pathway starts with FXa:FVa, known also as prothrombinase complex due to its ability to convert prothrombin (FII) into thrombin (42, 43) through cleavage of multiple peptide bonds, whose sequence may depend on the membrane source (44). The initially low amount of thrombin activates FV, FVIII, and FXI on platelets that become the cornerstone surface for further coagulation reactions (45–48).

After this initial sequence of events, coagulation is exponentially maximized for generation of massive amounts of thrombin, which reaches sufficient concentration to convert fibrinogen (FI) into fibrin monomers. Therefore, thrombin promotes its own additional generation during the “propagation” phase independently from TF:FVIIa complex. Indeed, on the platelet surfaces (membranes), FVIIIa binds FIXa, and activated FX (FXa) that will form additional FXa:FVa, whereas FXIa directly activates FIX that will form additional FIXa:FVIIIa complexes. Finally, the organized three-dimensional assembly of fibrin monomers in protofibrils and fibrin fibers produces the impermeable blood clot. In the coagulation cascade, cross-linking stabilization of the fibrin clot requires activated FXIII (FXIIIa), again produced by thrombin activity (49).

An additional amplification loop by thrombin promotes platelet activation and aggregation via the cleavage and activation of proteinase-activated receptors (PARs) (1). However, activated PARs may modulate various cellular activities under different pathophysiological conditions including inflammation, apoptosis, cell migration, angiogenesis and tissue remodeling (50). Notably, hemostasis components can elicit opposite signaling responses through activation of the same PAR, as provided by *in-vitro* evidence, where PAR-1 may induce pro-inflammatory and anti-inflammatory signaling under activation by thrombin or the anticoagulant activated protein C (aPC), respectively (51, 52). It has been demonstrated that under coagulant conditions, FXa binds PARs (PAR-1 and PAR-2) at the vascular endothelial cell level, evoking the production of proinflammatory cytokines IL-6 and IL-8 (53), and the monocyte chemotactic protein-1 (54). Subsequent thrombin production reinforces the signal already started by FXa, sustaining the production of the proinflammatory cytokine IL-8 through PAR-1 (53). In addition, FXa triggers a series of Ca^2+^ oscillations (53), which may have a function in the Ca^2+^-dependent activation of proinflammatory transcription factors (55). Moreover, FXa induces expression of adhesion molecules promoting leukocyte adhesion (54), which in turn may also be sustained by the co-localized presence of thrombin and fibrinogen (56, 57).

Based on these findings, it has been hypothesized that coagulation activation at the neurovascular interface might contribute toward eliciting and sustaining the inflammatory phenomenon characteristic of MS pathophysiology. This has been investigated to some degree, albeit insufficiently.

It has been established that some coagulation factors are expressed in the CNS, including FX and FII (58–61). However, the physiological functions related to their presence are mostly unknown. Depending on the degree of BBB damage, blood components (but not blood cells) like the high molecular weight fibrinogen as well as FV (62) can enter into the CNS, thus providing the complete repertoire of factors to trigger coagulation. Nevertheless, in order to form fibrin, a consistent amount of protein is needed, and in addition, an activated surface that sustains the coagulation process. As of now, the exact sequence of events that supports coagulation in the CNS and fibrin formation, in particular in MS patients, is inferred from the general coagulation pathway and does not take into account the specificity of astrocyte membranes.

Several findings in mice, and particularly in the experimental autoimmune encephalomyelitis (EAE) model, support the importance of coagulation factors in MS, either procoagulant in the extrinsic and intrinsic pathways, or anticoagulant. The key event in the CNS is the entry of fibrinogen, the leakage of which correlates with areas of axonal damage and has been shown to cause the undesired activation of microglia, subsequently inducing the recruitment and activation of macrophages, thus promoting inflammatory responses (6, 7). The fibrinogen enters into the CNS after BBB leakage and induces reactive oxygen species (ROS) release in microglia and its signaling via the microglial receptor CD11b^+^ is required for development of axonal damage in EAE (6). The first EAE-related work that described the role of fibrinogen in activating microglia/macrophages through specific interaction with the CD11b^+^/CD18 integrin receptor also showed protection either by genetic disruption of the fibrinogen region that contains the sequence for CD11b^+^ interaction or by pharmacological blockage of this fibrinogen region with an inhibitory peptide (7). Intriguingly, treatment in this animal model with recombinant thrombin (depleted of pro-coagulant function) significantly ameliorates the pathological condition, reducing inflammatory cell infiltration, and demyelination, decreasing activation of CD11b^+^ macrophages and reducing the accumulation of fibrin(ogen) in the CNS (63). This supports the idea that the pro-coagulant function of thrombin is involved in microglial activation (64).

Other experimental findings support the role of fibrinogen in suppressing remyelination by the inhibition of oligodendrocyte progenitor cell differentiation into myelinating oligodendrocytes (9). In the EAE marmoset model, fibrinogen was proposed to derive from the central vein in early lesions, and its deposition was found to precede demyelination and visible gadolinium enhancing lesions on MRI (65). In fact, the peak of fibrinogen deposition corresponded with the beginning of demyelination and axonal loss. Afterwards, fibrinogen was found inside microglia/macrophages, suggesting its phagocytosis. Moreover, a positive correlation between fibrinogen deposition and accumulation of microglia/macrophages and T cells was detected (65). Overall, fibrinogen leakage is one of the earliest detectable events in lesion pathogenesis. Very recent promising data in EAE mice have shown that a monoclonal antibody targeting fibrin, without interfering with the coagulant activity, avoids microglia activation, and monocyte infiltration into the CNS (66). Moreover, it decreases neurotoxicity through the inhibition of ROS production mediated by NADPH oxidase in the innate immune cells, which has been demonstrated to be fibrin induced during the neurodegenerative process (66).

### Fibrinolytic Pathways

The dissolution of the fibrin clot is mediated by the fibrinolytic system (Figure 2), initiated by the conversion of plasminogen into active plasmin by either urokinase-type plasminogen activator (uPA) or tissue-type plasminogen activator (tPA). tPA was found to be the most abundant plasminogen activator in control brains, with antigen concentration and enzyme activity several orders of magnitude higher than those of uPA (67). Plasmin cleaves fibrin to soluble degradation products, particularly the D-dimers, which represent an indicator of cross-linked fibrin turnover (68). Strikingly, components of the fibrinolytic system present in the CNS participate in a wealth of physiological roles (69).

tPA has been found to be involved in regulating cerebrovascular integrity (70), neuronal activity (through its action on the N-methyl-D-aspartate (NMDA)-receptor), neuronal calcium signaling, axonal regeneration, and microglial activation/inflammation (69). uPA exerts proteolytic and intracellular signaling functions by binding its receptor (urokinase plasminogen activator receptor, uPAR) on the cell surface, including microglial activation and axonal regeneration (71–73).

The activity of both tPA and uPA is regulated by specific plasminogen activator inhibitors (PAIs) of which the principal is PAI type 1 (PAI-1), a member of the serine protease inhibitor superfamily (SERPINS) (74). Tight connection of fibrinolysis with coagulation is further provided by thrombin, which enhances fibrinolysis that induces the expression and activity of tPA, and causes inactivation of PAI-1 by forming a complex with it. Interestingly, high PAI-1 expression may be induced by inflammatory cytokines in pathological conditions (75).

Experimental evidence in mice has demonstrated that PAI-1 can be released by microglia and astrocytes under inflammatory conditions, increasing microglial migration into the brain and inhibiting microglial phagocytosis (76). Accordingly, in EAE mice, the inhibition of PAI-1 has been shown to decrease axonal degeneration and demyelination (77). Conversely, tPA deficiency in EAE mice induces a more severe disease progression and CNS fibrin deposition, while uPAR depletion delays the disease onset, acting only in the initial stage by reducing the adhesion and migration of inflammatory mononuclear cells into the CNS (78). In fact, mice in the EAE model without uPAR subsequently develop chronic disease (78). Thus, data in animal models suggest that an impaired fibrinolytic pathway may be involved in both inflammatory and neurodegenerative processes of the disease.

### The Eclectic Nature of Factor XII: The Crossroad Between Coagulation (Intrinsic/Contact Pathway), Inflammation, and Immunity

Recently, albeit only in an animal model, FXII was found to be involved in adaptive immune responses via uPAR (CD87)-mediated modulation of dendritic cells (DCs) (10).

**Figure 3:**
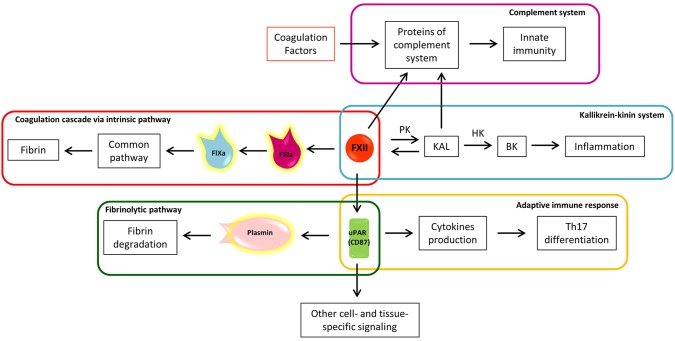
The eclectic nature of Factor XII: the crossroad between coagulation, inflammation, and immunity. a, activated; BK, bradykinin; F, factor; HK, high molecular weight kininogen; KAL, kallikrein; PK, prekallikrein; uPAR, urokinase plasminogen activator receptor; Th17, T helper 17 lymphocytes.

The coagulation cascade may be triggered by the circulating protein FXII, also called Hageman factor (79), through its contact with negatively charged surfaces and conformational change in the catalytic domain. The contact activation system does not depend on “external” proteins to trigger the coagulation cascade and it is usually identified with the intrinsic coagulation cascade pathway (80, 81). The FXIIa-initiated intrinsic coagulation pathway proceeds through activation of FXI (FXIa) and subsequent FIX activation (FIXa) (Figure 3), hence reaching the common pathway (Figure 1). Despite its contribution to fibrin formation in coagulation assays, the role of factor FXII “*in-vivo*” has long been debated because FXII deficiency does not exhibit a clinically relevant bleeding phenotype (82). Considering that FXII is located at the crossroads of several other pathways, these features make FXII an attractive target for inhibition without concomitant bleeding complications (83, 84).

FXIIa converts prekallikrein (PK) to kallikrein (KAL) (80), starting the proinflammatory kallikrein-kinin system (Figure 3). KAL acts on high molecular weight kininogen (HK), releasing the active peptide bradykinin (BK), through which bradykinin receptors mediate: (1) vasodilation induced by nitric oxide formation, (2) prostacyclin release, which reduces vessel-wall exposure of TF, (3) platelet inhibition, and (4) tPA release (80). The kallikrein-kinin system is further linked to the fibrinolytic pathway by KAL, which is able to convert plasminogen to plasmin (85). Thus, from one side the kallikrein-kinin system through BK promotes inflammation and from the other, the inhibition of coagulation and promotion of fibrinolysis. In the EAE animal model, the blocking of a BK receptor (B1R), mainly expressed close to plaques, prevented the infiltration of T lymphocytes into the CNS and decreased BBB permeability (86).

Interestingly, FXIIa itself has the capacity to cleave several proteins of the complement system, driving activation of innate immunity against foreign pathogens (Figure 3) (87). The complement cleavage products (C3a and C5a) have also been shown to exhibit robust chemo-attractive properties to human mast cells and neutrophils, highlighting the pro-inflammatory effects of the coagulation-complement interplay (88).

An example of selective pathway activation is given by mast cells (89) that rapidly secrete granules, of which heparin is one of the major constituents, when activated. Although heparin is primarily an anticoagulant, it provides a negatively charged surface that activates FXII, thus selectively promoting the inflammatory kallikrein-kinin system and possible consequent vascular leakage and BK-driven leukocytes infiltration (89).

Another immuno-mediated mechanism able to induce FXIIa is supported by neutrophils through the release of neutrophil extracellular traps (NETs). NETs consist of negatively charged contents such as nucleic acids together with histones, and antimicrobial proteins, which are physiologically used to trap and kill bacteria during infection. On the other hand, they trigger FXIIa and, in addition, foster the recruitment and activation of platelets, thereby promoting immunothrombosis (90).

In EAE, it has been demonstrated that depletion of FXII has a protective effect, delaying disease onset and decreasing disease severity (10). Of note, no differences were found in the amount of fibrin/fibrinogen in the CNS of EAE-FXII depleted mice compared to those with the wild-type EAE phenotype. Futhermore, factor XI (directly activated by FXII) deficiency does not alter the clinical course, demyelination, cytokine levels or the immune cell infiltration in the EAE model. These results support the hypothesis that FXII does not participate through activation of the intrinsic coagulation pathway, which would imply that the FXII procoagulant activity “*per se*” is not involved in MS (10).

### Platelets, Von Willebrand Factor, and ADAMTS13

Hemostasis is a complex multi-step process, involving the interaction of platelet adhesion receptors with cognate ligands such as von Willebrand Factor (vWF), collagen, and fibrin (1).

**Figure 4:**
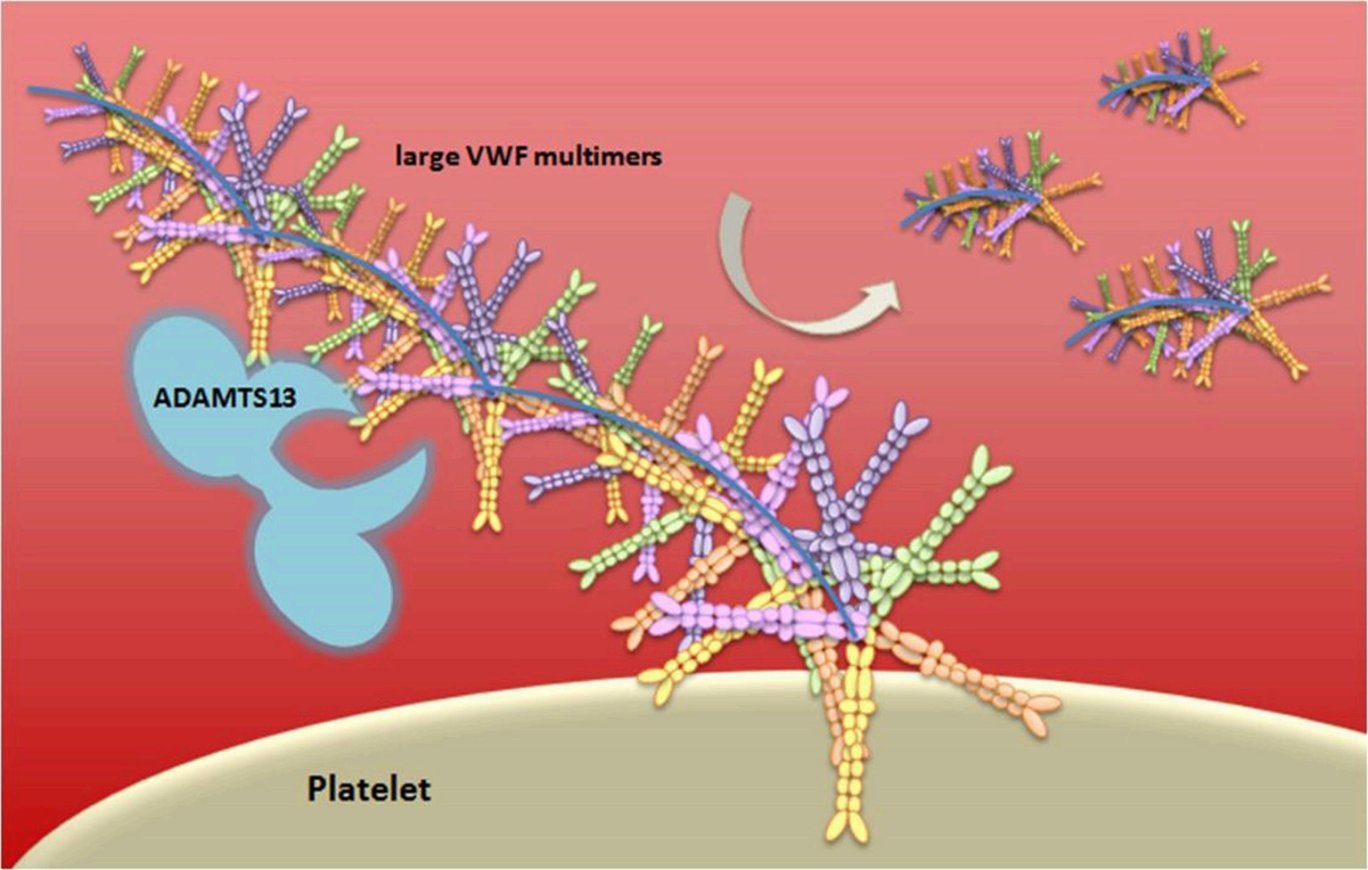
Schematic representation of vWF multimer size regulation by ADAMTS13. von Willebrand Factor (vWF) is stored in the Weibel-Palade bodies of endothelial cells or in the α-granules of platelets and it is released in an ultra-large form, a long multimeric string. The vWF serves as an adhesion surface to which platelets adhere and aggregate, and form a plug. The regulation of platelets adhesion depends upon cleavage of vWF in different size of multimeric string by ADAMTS13.

vWF is either constitutively produced or released by Weibel-Palade bodies from endothelial cells, stored platelets, and subendothelial connective tissue, in an ultra-large form, a long multimeric string that is associated with FVIII molecules (91). When thrombin cleaves FVIII, it mediates its activation through extended conformational changes that additionally cause FVIII to dissociate from vWF (92). vWF serves as an adhesion surface to which platelets aggregate and form a plug. The “A Disintegrin-like And Metalloprotease with ThromboSpondin type 1 motif 13” (ADAMTS13) enzyme, a main inhibitor of hemostasis, cleaves the ultra-large vWF in vWF multimers with lower size, decreasing the propensity of vWF to support platelet adhesion and aggregation (93) (Figure 4). Deficiency of ADAMTS13 causes thrombotic thrombocytopenic purpura (TTP), a disease characterized by overt platelet aggregation through large vWF multimers generating microvascular thrombosis (94).

### Coagulation Inhibitors

*In vivo*, coagulation factors are regulated by positive and negative feedback loops, the latter being provided by multiple coagulation inhibitors/anticoagulant proteins, which are also activated in a cascade-like fashion and influenced by feedback loops.

In the coagulation amplification process, the first line of inhibition is exerted by tissue factor pathway inhibitor (TFPI) (95), membrane-bound to endothelial cells (TFPIβ) as well as soluble in plasma, that is released from endothelial cells and platelets (TFPIα) (95). Circulating TFPI is mainly associated with lipoproteins, and inhibits coagulation in two distinct ways: (1) primarily, by interaction with the transient TF/FVIIa/FXa complex, and (2) by direct inhibition of free FXa. TFPI-dependent inhibition of FXa is mediated by the presence of protein S, which acts as a cofactor increasing TFPI affinity (96).

TFPIβ, which appears to be the predominant form, is anchored on the surface of the vascular endothelium, suggesting its role in the regulation of TF-mediated inflammatory responses via PARs signaling (97, 98). TFPI coagulation-independent action includes the suppression in TNF-α and IL-6 production, and an increase of anti-inflammatory IL-10 (99). Thus, TFPI may have distinct biological activities and potentially exerts a protective anti-inflammatory role in MS.

Antithrombin (AT, previously also called antithrombin III) belongs to the family of SERPINS and it inhibits several activated coagulation factors: FVIIa in complex with TF, FXa that dissociates from the TF-bearing cell (74) and, as the name suggests, thrombin via the formation of the thrombin-antithrombin (TAT) complex. Binding of cofactor heparin and heparin-like molecules are required for achieving inhibitory rates of AT.

The rising concentration of thrombin during coagulation proteolytically activates a main coagulation inhibitor, protein C, by binding the membrane protein thrombomodulin (TM), which is expressed on endothelial cells together with the endothelial protein C receptor (EPCR) (51). As with other receptors involved in hemostasis, both TM and EPCR can be cleaved from the cell surface in response to endothelial damage (100, 101). Activated Protein C (aPC) associates with its cofactor protein S, and the aPC/protein S complex proteolytically attacks FVa and FVIIIa, which are mostly membrane-bound, thus suppressing tenase and prothrombinase complexes (51). Of note, aPC may inactivate FVa when the thrombin-generating surface is provided by endothelial cells, but not from platelets (102). aPC may remain associated with EPCR and interact with PARs, exerting antiapoptotic and anti-inflammatory actions as signaling molecule (51), thus providing a tight link between (anti)coagulation and (anti)inflammation.

It has been observed in EAE that the administration of recombinant aPC and mutant forms of aPC with either the anticoagulant function alone or the signaling function alone reduces disease severity. This provides evidence that both of aPC's anticoagulant and signaling functions are required to improve the disease condition (103). EAE mice with a TM gene mutation that disrupts the TM-dependent activation of protein C (TM^Pro/Pro^) have perturbed myelination and mitochondrial functioning, resulting in increased ROS production and aggravated EAE pathology. Administration of aPC or TM provided relief in TM^Pro/Pro^ EAE mice (104, 105). Given these results, the role of the protein C system in amelioration of the disease is worth further consideration.

Other noteworthy inhibitors from the SERPINS family are heparin cofactor II (HCII), C1 inhibitor (C1INH) and protein C inhibitor (PCI). The inhibitory role of SERPINS is modulated by binding to cofactors, especially glycosaminoglycans like heparin, which present on cell surfaces and on the extracellular matrix (74, 106). HCII acts similarly to AT in the negative regulation of thrombin (107). C1INH is the most powerful FXIIa inhibitor (108). PCI inhibits anti-coagulant aPC and thrombin-TM complex but also the pro-coagulant thrombin, FXa, FXIa and FVIIa-TF complex. It also inhibits the fibrinolytic pathway by inhibiting uPA and tPA (74).

Overall, pro-coagulant, anti-coagulant, and fibrinolytic pathways are responsible for maintaining the hemostasis balance under physiological conditions. Significant deviation from these pathways would result in hypercoagulability leading to life-threatening thrombotic or, alternatively, to acquired/inherited bleeding diseases (e.g., hemophilias). Based on this, the role of coagulation (im)balance in MS patients is further reviewed below.

## Coagulation and Hemostasis Findings in Multiple Sclerosis patients

### Fibrin(ogen) Brain Deposition

Direct studies of histological brain samples, aimed at addressing fibrin deposition and alteration of the fibrinolytic pathway, began in the 1980s (Tables 1, 2). Nowadays, it is well known that one of the key events in the pathophysiology of MS is BBB breakdown, which leads to the entry of several neurotoxic blood-derived proteins (Figure 1) (119). Thanks to these histological studies, fibrinogen, an abundant protein in plasma, has been identified as a contributor to neuroinflammation in the CNS (11, 120). However, since most of the antibodies used across these studies were unable to distinguish fibrin from fibrinogen, the term “fibrin(ogen)” is likely more appropriate. The properties of fibrin favor the formation of oligomers and protofibrils, which aggregate laterally to make fibers, and ultimately branch to yield a three-dimensional network of insoluble fibrin (121). The detection in tissues of insoluble fibrin (fibrin deposition) by antibodies is therefore enhanced as compared with the detection of fibrinogen.

**Table 1:** Histopathological evidence of hemostasis components in multiple sclerosis.

**Hemostasis components**	**Main findings (patient sample size/methodology)**	**References**
**COAGULATION**		
FXII	Deposition nearby dendritic cells positive for uPAR.	(10)
Fibrinogen	Presence within demyelinated centers (23 acute MS plaques).	(109)
	Detected in 19 inactive plaques, co-localize with astrocytes (32 inactive plaques).	(110)
	Perivascular detection in type I, II and V lesions. Leakage within central plaques parenchyma (immunohistochemistry on 155 MS lesions from 13 early cases of MS).	(111)
	Extravascular staining with perivascular distribution in association with microglial activation (active MS lesions analyzed by confocal microscopy).	(112)
	Perivascular distribution of leakage and differential degree of deposition in WM. Co-localization with astrocytes. Correlation with the grade of tight junctions' abnormality (2,198 MS and 1,062 control vessels analyzed by confocal microscopy).	(113)
	Leakage both in active and chronic lesions; reactivity also in NAWM and in WM. Co-localization with astrocytes and neuronal process (postmortem MRI on MS lesions).	(2)
	Extravasation close to the blood vessels only in chronic active lesions (4 chronic active lesions and 5 chronic inactive lesions from 4 MS brains).	(65)
Fibrin(ogen)	Extracellular deposition predominantly located in layers 5 and 6 of the cortex in MS. Intracellular deposition detected in neurons and astrocytes (immunohistochemistry on the cortex of 47 progressive MS and 10 controls).	(12)
Fibrin	Staining overlap with macrophages and axons, and extended into NAWM (32 inactive plaques).	(110)
	Deposition in areas of activated microglia (immunohistochemistry on 155 MS lesions from 13 early cases of MS).	(111)
	Deposition occurs in pre-demyelinating areas of activated microglia.	(11)
**INHIBITORS**		
Protein C inhibitor	Detected in chronic active plaques (Mass spectrometry MS plaques).	(103)
C1INH	Detected in MS plaques.	(114)

*C1INH, C1 inhibitor; F, factor; NAWM, normal-appearing white matter; MS, multiple sclerosis; uPAR, urokinase plasminogen activator receptor; WM, white matter*.

**Table 2:** Histopathological evidence of fibrinolytic pathway components in multiple sclerosis.

**Fibrinolytic components**	**Main findings (patient sample size/methodology)**	**References**
Fibrinolysis	Higher fibrinolytic activity in plaques than adjacent NAWM.	(115)
tPA	Staining for infiltrated mononuclear cells in MS lesions and WM. Strong positivity of foamy macrophages in areas of demyelination and decline in chronic lesions.	(116)
	Co-localization with non-phosphorylated neurofilament and fibrin deposition in demyelinated axons.	(67)
	Decreased tPA activity in acute MS lesions. Decreased fibrinolytic activity in demyelinating MS plaques due to tPA/PAI-1 complex.	(117)
tPA receptors	Localization on macrophages, astrocytes. Increased in MS lesions compared to NAWM.	(118)
uPA, uPAR	Detected in acute MS lesions, expressed by mononuclear cells in perivascular cuffs and to macrophages in the lesion parenchyma. uPAR additionally detected in NAWM.	(67)
D-dimers	Localization on foamy macrophages and demyelinating axons.	(117)
PAI-1	Detected in acute MS lesions, expressed by mononuclear cells in perivascular cuffs and to macrophages in the lesion parenchyma.	(67)
	Up-regulation in progressive MS cortex but without an efficient fibrin degradation (immunohistochemistry on the cortex of 47 progressive MS and 10 controls).	(12)

*NAWM, normal-appearing white matter; MS, multiple sclerosis; PAI-1, plasminogen activator inhibitor 1; tPA, tissue-type plasminogen activator; uPAR, urokinase plasminogen activator receptor; WM, white matter*.

The relation between hemorrhage and demyelinating plaques was first considered by an early case report of 2 MS patients who developed CNS hemorrhage. It was suggested that the demyelinating event could contribute and set the stage for focal hemorrhages (122). However, over the course of the following years, the leakage of blood protein fibrinogen into the brain parenchyma was established as a potential marker of BBB damage (2, 113), and as a contributor to neurodegenerative events. In initial reports, the presence of heavy extracellular fibrinogen was detected in demyelinated centers of acute MS plaques (109) as well as in most of the examined inactive plaques, particularly close to astrocytes (110). Moreover, the fibrinogen within the plaques was found to overlap with macrophages and axons, and even extended into the surrounding normal-appearing brain tissue. Nevertheless, fibrinogen did not co-localize with the enlarged astrocytes outside the plaques (110). Moreover, it was shown that fibrinogen leakage gradually increased through the progression of MS lesions, reaching the highest levels within the central parenchyma of those plaques with the greatest degree of activity (111). Interestingly, fibrinogen co-localized with areas of activated microglia in MS lesions (111).

Confocal microscopy confirmed the presence of extravascular fibrinogen in active MS lesions, most commonly with a distinct perivascular distribution, and in a few cases widely distributed throughout the parenchyma (112). Association of such leakage with areas of microglial activation was found to be consistent with increased tight junction abnormality in the same areas (112). Confocal microscopy was also extensively used to confirm the perivascular distribution of the fibrinogen leakage and demonstrate varying fibrinogen levels within MS lesions (113). Hence, the severity of altered tight junctions was associated with BBB dysfunction, which in turn was proportional to the increase in fibrinogen leakage reaching particularly high levels in active lesions (113). A threshold of tight junction injury might be required before significant and visible BBB leakage of the large, high-molecular-weight protein fibrinogen (113), which could explain the lack of fibrinogen detection close to vessels with a lower degree of tight junction abnormality.

Postmortem magnetic resonance imaging (MRI) was also applied to detect both diffuse and focal brain abnormalities, allowing targeted histopathological examination of MS lesions (2). BBB disruption was detected by increased immunopositivity for fibrinogen in the brain parenchyma as described by previous studies (109–113). Fibrinogen leakage was found in both active and chronic MS lesions, co-localizing with astrocytic processes and occasionally with axonal processes (as demonstrated by neurofilament immunoreactivity), which suggested that astrocytic and neuronal processes may bind or incorporate extravasated fibrinogen. Moreover, fibrinogen was not limited only to demyelinating lesions, but it was seen in both reactive lesions characterized by small clusters of microglial cells without apparent loss of myelin with a variable degree of edema, and in areas with diffusely abnormal white matter (WM) (2). Nevertheless, the presence of fibrinogen was more extensive in chronic active and inactive lesions when compared to reactive lesions (2).

A more recent analysis of chronic MS lesions revealed that fibrinogen extravasation was present in chronic active lesions close to the blood vessels, but not in the chronic inactive ones (65). It was also shown that fibrin deposition might occur early in MS and precede demyelination (11), since the “pre-demyelinating” areas of activated microglia hosted fibrin precipitates within the extracellular space of the lesions (11). The high precipitation of fibrin on the surface of microglia was suggested to be the driving force for microglial activation according to its detection in focal plaques of microglial activation with features of hypoxia-like damage but in the absence of demyelination (11). Thus, changes in the NAWM precede the formation of inflammatory demyelinating plaques, in particular in those exhibiting a pattern of hypoxia-like demyelination. Such changes were suggested to settle the inflammatory response and infiltration of T-cells, B-cells, and macrophages in the brain tissue, leading to the formation of the classic inflammatory demyelinating plaque detected by MRI (11). This is in agreement with recent findings showing that fibrin can mediate microglial activation and oxidative stress with ROS production, contributing to local neurodegenerative events (66). Finally, fibrin(ogen) was reported in the cortex of progressive (P-MS) cases. Extracellular fibrin(ogen) deposition was mostly found in the deeper cortical layers (layers 5 and 6 vs. layer 2). In contrast, its co-localization within neuritic and astrocytic processes was predominantly in the superficial cortical layers (12). The presence of intracellular fibrin(ogen) has been suggested to occur by direct synthesis of those cells or to be mediated by retrograde transport in damaged axons exposed to increasing amounts of protein. Overall, severe fibrin(ogen) deposition was detected in areas of significantly reduced neuronal density and particularly appeared to affect the loss of layer 5 projection neurons (12). No relationships were observed between the presence of fibrin(ogen) and microglial/macrophage density. Of note, the deposition of other proteins, such as albumin, remains controversial because of their inability to be converted into an insoluble matrix as fibrinogen does to fibrin, precluding accurate assessment.

In summary, these data point toward the role that fibrinogen has on sustaining the pathogenesis of MS lesions following its entrance into the CNS. In particular, its conversion into fibrin seems to trigger the activation of microglia and to support inflammation and the consequent development of demyelinating lesions.

### Histological Evidence for an Altered Fibrinolytic Pathway in Multiple Sclerosis CNS

Besides fibrin(ogen), several studies have focused on the fibrinolytic pathway (Table 2), and the capacity of MS lesions to break down fibrin. Initial findings were provided by histochemical techniques, showing that the amount of fibrinolytic activity was comparable between active lesions and inactive ones (115). The fibrinolytic zones in MS brains appeared to originate from areas around vessels or capillaries, and the presence of lymphocytic infiltrates, gliosis, or macrophages did not change the localization and degree of fibrinolysis. Moreover, the NAWM from MS patients was not more fibrinolitically active than that of the controls, but plaques showed more fibrinolytic activity compared to adjacent NAWM (115), suggesting to an attempt to combat fibrin. Subsequently, positive infiltrating mononuclear cells stained for tPA were observed in MS lesions, particularly within active ones (116). This pattern converted into a strong positivity of foamy macrophages in areas of demyelination and declined in chronic lesions. Similarly, PAI-1 expression paralleled that of tPA on foamy macrophages (116). The disappearance of immunoreactivity for tPA in chronic MS plaques also supported the role of impaired fibrinolysis as a contributing event to the inflammatory stage of demyelination mediated by fibrin. In fact, the increased expression of tPA on mononuclear cells in perivascular cuffs was suggested to be one of the earliest detectable signs of inflammation in MS. tPA might trigger the matrix metalloproteinase (MMP) cascade and thus facilitate entry of leukocytes into the CNS (116). It is important to note, though, that another study provided partially discordant data: although quantitatively decreased in MS lesions, it found that tPA was co-localizeed with non-phosphorylated neurofilament and fibrin(ogen) deposits on demyelinated axons (67). On the other hand, highly significant increases in uPA, uPAR, and PAI-1 were detected in acute MS lesions and uPAR in NAWM when compared to control tissue. These three proteins were immunolocalized with mononuclear cells in perivascular cuffs and with macrophages in the lesion parenchyma. The significant increase in the uPAR complex was thought to be a trigger for focal plasmin generation and for cellular infiltration, cooperating with MMP activity in the opening of the BBB (67).

Further investigations provided evidence for the lowest fibrinolytic activity within acute lesions, which was due to the formation of tPA/PAI-1 complex (117), in turn contributing to fibrin accumulation. Nevertheless, D-dimers and fibrin degradation products were mostly localized at the neurovascular interface and on foamy macrophages and axons during the chronic inflammatory stage of lesions (117). In addition, increased PAI-1 synthesis leading to defective fibrinolysis appeared to develop before lesion formation (117). However, during lesion progression, an increase in lower molecular weight PAI-1 peptides was detected, as a result of PAI-1 intracellular degradation mediated by macrophages (117).

Plasma membrane tPA receptors, which may concentrate proteolytic activity on the cell surface and in turn locally enhance the fibrinolytic response, were immunolocalized in acute MS lesions on macrophages and astrocytes (118) and increased in MS lesions when compared to NAWM samples. Furthermore, a tPA receptor was found on neuronal cells within the cortex. However, the limited availability of tPA, bound to PAI-1, reduces the production of plasmin, which further decreases the fibrinolytic activity in active MS lesions and increases axonal fibrin deposition and neurodegeneration (118). Indeed, perturbed fibrinolysis was found to be a hallmark of P-MS cases with abundant cortical fibrin(ogen) deposition (12). Overall, significant upregulation of PAI-1 in the cortex, where fibrin deposition was most severe, points toward dysregulated fibrin clearance that allows for its pathological accumulation in the later stages of MS (12).

### Detection of Protein C Inhibitor (PCI), C1INH, and FXII in Multiple Sclerosis Plaques

An early biochemical study based on isolation of brain capillaries from human brain samples close to MS lesions showed positive staining for FVIII (123). Further insights into coagulation components and inhibitors in MS lesions have been provided by lesion-specific proteomic profiling (103), which detected TF in particular. This is to a certain extent expected in relation to the abundance of this protein in perivascular spaces, whereas PCI is only found in chronic active lesions. PCI, which inhibits aPC, seems to accumulate within these lesions secondary to the disruption of the BBB during neuroinflammation. The combined presence of TF and PCI suggests pro-inflammatory thrombin formation and suppression of the PC pathway, supporting a mechanism involved in MS lesion formation that suppresses the action of coagulation inhibitors in the presence of coagulation activation (103). Further evidence for the intricate connection between coagulation, inflammation, and immunity was provided by the positive reactivity of MS lesions for proteins of the complement system, and regulators as C1INH. Taken together, these findings point toward continuing local complement synthesis, activation, and regulation despite the absence of evidence of ongoing inflammation (114). Interestingly, deposition of FXII, which is inhibited by C1INH and might support autoimmunity, was detected in the histological analysis of CNS tissue from MS patients nearby DCs positive for CD87 (uPAR) (10).

Overall, impaired fibrinolysis seems to reinforce fibrin(ogen)-associated damage in MS. Impaired inhibition of coagulation, and the contribution of coagulation factors through inflammatory and autoimmunity pathways in CNS therefore deserves further investigation.

### Historical Perspective of Hemostasis Abnormalities and Circulating Hemostasis Component Levels in Multiple Sclerosis

The first description of hemostasis abnormalities in MS was provided by Putnam (124) who reported the presence of definite thrombi in half of the analyzed MS cases (9/17). Thrombi were described as the frequent occurrence of perivascular hemorrhages within acute lesions and as a vascular obstruction in chronic lesions. Therefore, the primary abnormality of MS was suggested to reside in the alteration of the blood clotting mechanism (124), and as a consequence of this hypothesis, 43 MS cases were treated with dicoumarine for a timeframe between 6 months and 4 years (125). Despite the side effects, Putnam and colleagues concluded that anticoagulant treatment reduced relapses in the relapsing-remitting (RR) form of MS while the course of chronic progressive disease was not affected (125). Soon after, Putnam interpreted venous thrombosis as a possible pathognomonic process in MS, while others reported increased capillary fragility (126) and subcutaneous hemorrhages (127).

Later on in 1955, Persson reported increased levels of plasma fibrinogen in MS patients during relapse exacerbations, which were not related to thrombus formation (128). By that time, it was already known that fibrinogen levels were higher than in controls in the majority of chronic and degenerative diseases, thus laying the foundations for later discoveries of fibrinogen levels as a marker of inflammation (128, 129). A few years later, another study investigated blood coagulation in 33 MS patients and corroborated previous findings, showing no tendency toward increased blood coagulability (130). Overall, in the majority of investigated patients fibrinogen levels were within the normal range, despite wide variations that were not associated with the stage of the disease (130). A subsequent study in 10 MS patients explored both blood and cerebrospinal fluid (CSF) and revealed that neither had thromboplastin activity, nor significant abnormalities in blood platelet, coagulation factors, serum platelet-like activity nor fibrinogen levels (131). Despite the lack of abnormal findings, increased capillary fragility was reported (131).

The “antithrombic” activity of normal and pathological CSF was later discovered in 1961 (132). With the exception of larger proteins like fibrinogen and FV, further studies demonstrated the presence of coagulation proteins in the CSF (133) and corroborated findings that degradation products of fibrin were present under pathological conditions (62).

The discrepancy in results regarding coagulant balance of that epoch needs to be interpreted in light of possible unstandardized examination techniques. However, after 80 years from the first report on altered coagulation in MS (124), findings on this research topic in MS are still controversial. This is probably also due to the inclusion of small cohorts of patients and the sporadic analysis of circulating levels of certain hemostasis components, which prevents the consolidation of conclusions and clearly highlights the need for larger, more well-controlled additional investigations. Evidence regarding CSF, plasma and serum levels of hemostasis components is summarized in Table 3. Of note, levels are often used to refer to either protein concentration or activity of a protein without a clear distinction. However, concentration levels provide information that is independent of the protein's ability to be intrinsically functional and do not depend on activatory or inhibitory molecules. Similarly, testing the functional activity does not provide direct information about its protein concentration but integrates the influence of activators or inhibitors. During a PT or aPTT assay, information about clotting time is obtained, providing the overall functionality of the system. When alteration in clotting time is observed, it is possible to supplement the assay using a plasma depleted of a specific coagulation component (thought to be the cause of the alteration) in order to assess the specific functional activity of that component. Since ethylenediamine-tetra-acetic acid (EDTA) removes calcium from the sample, which is needed for blood clotting, plasma collected in EDTA is not an appropriate sample to test PT and aPTT.

**Table 3:** CSF, plasma, and serum evidence of altered hemostasis components in multiple sclerosis.

**Hemostasis factors, inhibitors, and receptors**	**Main findings (patient sample size/methodology)**	**References**
**CSF**		
Fibrinogen	Lower levels in CIS vs. PMS (proteomic profile by mass spectrometer in 24 CIS, 16 RRMS, 11 PMS).	(134)
TM	Higher levels in OIND vs. SPMS. Ninety percent of TM in CSF is related to intrathecal synthesis (17 relapse, 11 remission, 11 SPMS, 19 OND, 15 OIND).	(135)
**PLASMA**		
FII, FX, Fibrinogen, PC, FII, FX, FXI	Higher FII:c and FX:c in RRMS and SPMS vs. controls. No differences in activity of Fibrinogen, FXI and PC (PT in citrate plasma: 116 RRMS, 10 PPMS, 73 SPMS, 20 controls).	(136)
FXII	Higher FXII:c in RRMS and SPMS vs. controls. Higher activity correlates with higher occurrence of relapses and shorter relapse-free period (aPPT in citrate plasma: 138 RRMS, 13 PPMS, 90 SPMS, 19 CIS, 130 controls).	(10)
	Increased of FXII protein concentration levels and reduced function in MS (aPTT and ELISA on citrate plasma: 12 RRMS, 34 SPMS, 28 PPMS, 49 controls). Intrinsic thrombin generation in 10 PMS, 10 controls.	(137)
FXII, ADAMTS13, HCII, TFPI, TM	Lower ADAMTS13 levels in MS vs. controls. Higher TFPI levels in PMS vs. RRMS and vs. controls. No differences in FXII and HCII (ELISA on plasma EDTA: 85 RRMS, 53 PMS, 42 controls).	(138)
FII	Prothrombotic state in RRMS (thrombin generation on citrate plasma: 15 RRMS, 15 PPMS, 19 controls).	(139)
Fibrinogen	No differences in fibrinogen levels, PT and aPTT times (42 RRMS and 31 controls).	(140)
	High levels, particularly associated with active lesions on MRI (17 out 58: 45 CIS, 12 RRMS, 1 PMS).	(141)
vWF, TM	Higher vWF activity in active MS. No differences in TM protein concentration (26 RRMS, 35 controls).	(142)
AT	No differences in AT:c (37 RRMS, 32 SPMS, 34 controls).	(143)
EPCR	Trend for higher levels in MS (63 MS, 20 controls).	(144)
**SERUM**		
FX, Prothrombin, C1INH, FXIII, Plasminogen	Reduction of FX, prothrombin and C1INH levels in pre- and post-symptomatic MS serum. Reduction in FXIII and plasminogen in post-symptomatic MS (Mass spectrometry (pooled serum of 100 MS vs. pooled serum of 100 controls).	(145)
TM	Higher levels in MS during exacerbation vs. remission state, OND, and controls (17 acute relapse, 9 PMS, 13 HAM, 10 non-HAM, 10 OND, 20 controls).	(146)
	Higher levels in OIND vs SPMS (17 relapse, 11 remission, 11 SPMS, 19 OND, 15 OIND).	(135)
TM, aPC	No differences (100 RRMS, 22 SPMS, 122 controls).	(147)
vWF	No difference (9 RRMS, 9 SPMS, 10 PPMS).	(148)

*ADAMTS13, A disintegrin-like and metalloprotease with thrombospondin type 1 motif 13; AT, antithrombin; aPC, activated protein C; aPTT, activated partial thromboplastin time; C1INH, C1 inhibitor; CIS, clinically isolated syndrome; CSF, cerebrospinal fluid; :c, activity; EDTA, ethylenediamine-tetra-acetic acid; ELISA, enzyme-linked immunosorbent assay; EPCR, endothelial protein C receptor; F, factor; FII, thrombin; HAM, HTLV-1-associated myelopathy; HCII, heparin cofactor II; OIND, other inflammatory neurological disorders; OND, other neurological diseases; PC, protein C; PMS, progressive multiple sclerosis; PPMS, Primary Progressive Multiple Sclerosis; PT, prothrombin time; RRMS, relapsing-remitting multiple sclerosis; SPMS, secondary progressive multiple sclerosis; TFPI, tissue factor pathway inhibitor; TM, thrombomodulin; vWF, von Willebrand Factor*.

Considering the tight relation between coagulation factors and immune response discussed earlier, it is intriguing to speculate that the clinical manifestation of MS could also be related to increased pro-coagulant activity. No significant differences have been reported for PT or aPTT times in the plasma of MS patients (140) nor for fibrinogen concentration in either CSF or blood (140, 149). However, the analysis of the CSF proteomic profiles in patients, collected in different phases of their clinical course, showed significantly lower fibrinogen concentration in clinically isolated syndrome compared to PMS patients (134). Moreover, increased fibrinogen beta chain concentration was detected in CSF samples from two fulminant MS cases by mass spectrometry (150). A relationship with the activity of the disease was also reported in a recent investigation where high fibrinogen levels were detected in plasma in a substantial proportion (17/58) of patients, particularly in those with active lesions on MRI (141). Taken together, these studies support the role of fibrinogen as a contributor of neuroinflammation and neurodegenerative processes in the CNS following BBB damage.

In a more comprehensive investigation, the activity of PC, FII, FX, FXI and FXII, and propensity of fibrinogen to clot was determined in plasma samples of MS patients with different clinical phenotypes compared to healthy individuals (136). Increased activity of FII:C and FX:C was detected in RRMS and SPMS patients when compared to controls (136). These experimental findings suggest an increase in thrombin activity and its generation through FX activity, which by definition is part of the prothrombinase complex, in MS patients. However, these increased activities do not seem to be balanced by increased activity of PC, a key inhibitor (136). Similarly, plasma AT activity was reported to show no differences in MS patients or associations with periods of relapses or remissions (143).

Evaluation of coagulation activity by thrombin generation assay, a more sensitive and flexible method that accurately reflects the initiation, propagation, and termination phases of coagulation (151, 152), showed enhanced thrombin generation in RRMS patients compared to PPMS and controls, pointing to a prothrombotic state within the RRMS phenotype (139).

Elevated FXII activity was found in RRMS and SPMS compared to controls, and greater activity levels were associated with higher occurrence of relapses and shorter relapse-free periods, independently from the use of immune modulatory therapy (10). Another recent study that evaluated the ratio of FXII activity and the amount of circulating protein found increase activity of FII and FX was protein levels and reduced function within the intrinsic coagulation pathway (137). Notably, intrinsic thrombin generation did not result in the detection of prothrombotic features in the evaluated PMS patients (137). These data underline the importance of evaluating the activity of both antigen and coagulation factors. Furthermore, the contribution of FXII in MS may be independent of its coagulant property and the protein may potentially be hijacked to participate in other FXII-mediated pathways. Immune-modulatory function in relation to, or its possible independence, from coagulation activity, particularly for FXII, still remains to be elucidated in MS. On the other hand, in evaluating the activity of cellular components of coagulation, unstimulated and stimulated monocytes were not found to differ in MS and controls with respect to expression of cell surface TF or production and secretion of TF (153), which does not support the presence of pro-thrombotic components on cell surfaces. However, higher TFPI levels in P-MS patients compared to RR-MS patients and controls were recently reported (138). Taking into account that TFPI is the first line of inhibition in the coagulation cascade, this could merit further investigation.

Analysis in pre-symptomatic and post-symptomatic MS pooled serum detected proteomic changes for factors involved in the complement and coagulation pathways, with a particular decrease in MS of FX, FII, and C1INH (145). Because the serum is isolated after coagulation, these results might be interpreted as residual coagulation factors remaining after conversion of fibrinogen into fibrin, the last step of the pathway. Concerning the complement protein, lower C1INH in patients is of interest in light of its inhibitory activity against FXII through its recruitment in the CNS. Although depositions of FXII and C1INH have been reported, the demonstration of their co-localization in MS brain tissue is still needed (10, 123).

It is worth mentioning that a few studies have investigated platelet stickiness in MS (154–156). Nevertheless, platelets adhesion and aggregation are supported by vWF activity, which was found to be higher in patients with active MS than in controls, and significantly decreased after immunosuppressive treatment (142). The authors suggested that vWF could serve as a marker for evaluating BBB breakdown resulting from endothelial damage in MS, in line with the idea of hemostasis activation at the neurovascular interface after injury. Interestingly, decreased ADAMTS13, which exerts an inhibitory function on vWF activity, was detected in MS patients and in particular in those with cerebral microbleeds (138, 157). These data should motivate future investigations in MS patients with focal extravascular leakage of blood components, measured as cerebral microbleeds, to potentially identify as of yet unknown molecular component(s) driving hemostasis alteration in MS patients.

In addition to vWF release as consequence of endothelial damage, the shedding of membrane proteins could be promoted. In this context, soluble TM has been the most investigated protein in MS (135, 138, 142, 146, 147). Studies on larger cohorts of MS patients concluded that TM concentration levels do not differ in plasma or in serum (138, 147). However, circulating TM appeared to increase in MS during exacerbation compared to the remission, reaching higher concentration levels in patients with acute relapse (146). An intriguing hypothesis was also offered by the authors of a study who speculated that 90% of TM in CSF is of intrathecal synthesis, with higher TM production during relapses (135). Based on this hypothesis, TM should be further explored in association to the relapsing and remitting states of patients.

Correlations between peripheral plasma levels of FXII, TFPI, ADAMTS13, HCII, and TM with MRI measures, indicative of severity of inflammatory and neurodegenerative tissue injury, were recently investigated for the first time (138). Several correlations were detected in MS patients: (1) higher FXII levels with lower ventricular and higher deep gray matter (DGM) volumes, (2) higher HCII levels with lower brain and cortical volumes and higher ventricular volume, (3) higher TFPI levels with lower DGM volume. However, after correction for multiple comparisons, no significant relationships between hemostasis component levels and MRI measures remained significant. Nevertheless, in light of the fact that glycosaminoglycans are cofactors of HCII, and considering their role in the inflammatory process (158), as well as in normal CNS functioning or pathological conditions [MS included, as reviewed in reference (106)], the study of HCII involvement in MS deserves further investigation.

Overall, the discordant data on prothrombotic features in MS patients in the limited number of studies point toward altered pro-coagulant status during the more active phase of the disease. Patient prothrombotic heterogeneity could be approached through stratification according to coagulation balance in order to prospectively evaluate the impact of coagulation differences on disease evolution.

### Plasma, Serum, and Cerebrospinal Fluid Levels of Fibrinolytic Pathway Components

Early evidence of increased fibrinolytic activity, which again pointed toward an altered coagulation system in MS (159), and evidence of fibrin degradation products in the CSF of MS patients (160) paved the way for further studies investigating the proteins of the fibrinolytic pathway (Table 4).

**Table 4:** CSF, plasma, and serum evidence of fibrinolytic pathway components in multiple sclerosis.

**Hemostasis components**	**Main findings**	**References**
PAI-1	Higher levels in MS vs. controls. PAI-1 concentration has a reverse relationship of tPA:c (ELISA in CSF and plasma EDTA of 19 MS, OND, controls).	(161)
	Higher levels in MS vs. controls (ELISA on plasma EDTA: 85 RRMS, 53 PMS, 42 controls).	(138)
PAI-1, tPA	High levels of PAI-1 during relapses. No differences for tPA. No correlation between PAI-1 CSF and plasma levels (Plasma of 12 active RRMS, 12 stable RRMS, 10 controls).	(162)
tPA	Higher activity in MS (CSF of 7 MS, 9 leukemia, 21 encephalitis, 20 controls)	(163)
PAI-1, tPA, D-dimer	No differences (Plasma of 90 MS, 250 glioma patients 270 controls).	(164)
D-dimer	Higher levels in MS (ELFA on plasma of 42 RRMS, 31 controls).	(140)

*CSF, cerebrospinal fluid; :c, activity; EDTA, ethylenediamine-tetra-acetic acid; ELISA, enzyme-linked immunosorbent assay; ELFA, Enzyme Linked Fluorescent Assay; OND, other neurological disorders; PAI-1, plasminogen activator inhibitor 1; PMS, progressive multiple sclerosis; RRMS, relapsing-remitting multiple sclerosis; tPA, tissue-type plasminogen activator*.

In the CSF, higher tPA activity was found in MS patients (163) coupled with evidence of very low uPA activity (163). Increased total PAI-1 concentration was found in MS patients and a significant inverse relationship between PAI-1 levels in CSF and plasma was observed (161). Very high PAI-1 levels were observed during relapses (reaching values 6 times greater than controls), and follow-up investigation showed 2-fold decreased values even 1-2 months after the relapses. However, a correlation between PAI-1 and tPA plasma levels was not observed (162).

Recently, higher PAI-1 plasma levels in MS patients were reported when compared to controls (138). Moreover, in MS patients, but not in healthy controls, a positive association between PAI-1 and FXII concentrations and a negative association between PAI-1 and HCII concentrations were found. Accordingly, if thrombin is inhibited, there is no fibrin formation, hence there is no fibrinolysis to block. On the other hand, FXII stimulates fibrinolysis through uPAR (Figure 2), and if tPA increases, an inhibition target exists for PAI-1.

Again in plasma, levels of D-dimer, tPA, and PAI-1 did not differ between patients and controls in one study (164), but significantly higher D-dimer levels were found in another investigation (140).

Overall, data regarding increased PAI-1 antigen levels supports the notion that impaired fibrinolysis sustains the ongoing neuroinflammatory (particularly during relapse) and neurodegenerative events in the brain as evidenced by histological studies. Considering the few and discordant studies on tPA and D-dimers, further investigation is needed, which would provide a more comprehensive view of fibrinolysis in relation to the MS disease course.

### Effect of Disease-Modifying Treatments on Coagulation Pathways

Disease-modifying treatments (DMTs) are potential modifiers of coagulation factor levels. However, few studies are available on this topic. MS patients treated with steroids showed lower plasminogen and fibrinogen levels (165). Additionally, increased fibrinolytic activity has been observed in treated MS patients. At the time, these abnormalities were considered to be a consequence of a non-specific activation of coagulation in a setting of chronic immunological disease (165). However, because of the aforementioned role of fibrinogen and potentially decreased fibrinolysis in MS, these data are of interest and deserve additional investigation.

Another study investigated patients that developed progressive multifocal leukoencephalopathy (PML) under natalizumab treatment (pre-PML) (166). PAI-2, uPA, uPAR, TFPI, and TM were among the top differentially expressed genes in peripheral blood mononuclear cells collected at baseline and during PML. These genes were significantly down-regulated at baseline in pre-PML patients compared to the group that did not develop PML, providing evidence for a potential role of natalizumab as a modifier of hemostasis component levels. Although levels of serum proteins encoded by the differentially expressed genes did not show significant differences (166), their evaluation in plasma would be potentially informative.

Because glucocorticoids induce procoagulant reactions, the effect of high-dose intravenous methylprednisolone on fibrinogen, FVIII activity, vWF protein concentration, TAT, prothrombin fragments1+2 (F1+2), tPA protein concentration, PAI-1 activity and plasmin-antiplasmin complexes (PAP) was investigated using a prophylactic low dose of low molecular weight heparin (167). Whereas, the fibrinogen levels significantly decreased, FVIII activity and vWF protein concentration significantly increased in the absence of evidence for fibrinolytic system activation or suppression (167). At high-dose methylprednisolone, 5 out of 188 MS patients developed venous thrombosis, which led the authors to speculate on the synergistic effect between the treatment and MS immunopathology (168), which could predispose patients to prothrombotic risk.

In RRMS patients under glatiramer acetate (GA) treatment, TM levels were significantly increased compared to the respective drug-free group and healthy controls, regardless of the presence of current relapse. The authors speculated about a GA-induced mechanism of neuroprotection potentially leading to the generation of aPC (144).

A recent study on this topic evaluated EDTA plasma levels of a number of coagulation inhibitors (TFPI, ADAMTS13, HCII, TM, and PAI-1) according to DMT status, particularly in patients being treated with interferon-beta (IFN-b) or GA, each used in 1/3 of the patients. The high overall variability of levels in patients, and particularly those of PAI-1, could have prevented the detection of significant differences in the circulating levels of these proteins, including TM (138).

Considering the heterogeneity of coagulation balance in MS patients and the few studies that evaluated the effect of DMTs on coagulation, prospective investigation would be of great help to develop a better understanding drug–hemostasis interactions.

### Case Reports of Autoimmunity Affecting Hemostasis in Multiple Sclerosis

Unfortunately, very few cohort studies addressed whether or not coagulation imbalance was supported by the immune activity. Higher frequency of antiphospholipid antibodies, belonging to the IgM family, were observed in MS patients during exacerbation (10 out of 17 patients, 2–4 fold increase) compared to remission. Of note, a significant correlation between contrast-enhancing lesions and antibodies against FVII was found (169).

Case reports of autoimmunity affecting hemostasis components in MS shold be considered in light of the acquired dysregulation of coagulation, with a focus on those components that are mainly targeted and that may contribute to clinical worsening. Interestingly, a few cases have been reported with TTP episodes (170) and acquired ADAMTS13 deficiency in the context of IFN-b treatment for MS (171, 172). Despite the limitation of their low number, these reports highlighted acquired deficiency induced by auto-antibodies against ADAMTS13. Notably, lower levels of ADAMTS13 have been reported in MS compared to control subjects (138, 157). Additionally, several MS patients who received alemtuzumab treatment developed autoimmune TTP (173, 174).

Analogously, but on the other side of the spectrum, anticoagulant FVIII inhibitors may arise in autoimmune diseases, during and after pregnancy, and during drug therapy including IFN-alpha (used to treat leukemia and blood disorders such as TTP) with the outcome of acquired severe hemophilia. The first case report of an MS patient who developed hemorrhagic disorder was described as a rare case of antibody development against FIX and FVIII (175). A second case of acquired FVIII inhibitor was later described (176), and an additional case was reported in an MS patient after IFN-b treatment (177). Acquired hemophilia has also been described as an extremely rare complication in patients treated with alemtuzumab. Other case reports in the literature include two sisters with MS who had a quantitative deficit of factor VIII-vWF complex (178). These phenomena could be mediated by secondary B cell-mediated autoimmune complications leading to inhibitory autoantibodies to coagulation FVIII (179). However, a reference study for thrombophilia reported that among the 4,311 patients with a first episode of venous thrombosis, 30 had MS with increased FVIII activity levels (180).

Imbalance of the coagulation system seems to be supported by inflammatory and immune activity (181). Taken together, these reports and the role of (auto)immunity in MS, clearly support the need for additional investigation of specific acquired autoimmunity affecting hemostasis factors, either with pro-coagulant or anti-coagulant outcomes.

## Conclusions and Future Directions

The data reviewed indicate that fibrinogen leakage due to BBB damage in MS is consistent with microglial activation, particularly once thrombin cleaves it to fibrin. Additional fibrin(ogen) deposition might occur early in MS and precede demyelination, as well as contribute to cortical pathology in the progressive stages. Although histological studies point to fibrin(ogen) as a principal contributor to neuroinflammation and neurodegeneration in MS, these processes are also supported, and may be potentiated, by decreased fibrinolysis. In particular, increased PAI-1 synthesis and decreased tPA activity in MS lesions reflect an impaired clearance of fibrin due to the formation of tPA/PAI-1 complex, further contributing to the inflammatory stage of demyelination.

Other mechanisms also contribute to this intricate pathophysiological picture. The combined presence of TF and PCI in MS lesions suggests pro-inflammatory thrombin formation and suppression of the anti-inflammatory aPC pathway. Moreover, deposition of FXII might support autoimmunity through increased expression of uPAR, which has been reported in MS lesions. At the peripheral level in MS, coagulation activity variation still remains to be elucidated, and underlying factors uncovered. As potential contributors to the heterogeneity, protein plasma levels of hemostasis factors have been sporadically investigated in MS, and some results suggest that they could serve as potential biomarkers of ongoing alterations in the CNS. Indeed, the lower ADAMTS13 levels detected in MS patients (and in particular in MS patients with cerebral microbleeds) should stimulate further investigation based on potentially enhanced vWF activity in plasma, which has been suggested as a possible marker for evaluating the endothelial damage, leading to BBB breakdown.

Additional experimentation by use of high throughput transcriptomic and proteomic techniques is needed to determine how hemostasis components contribute to or decrease inflammatory and immune responses in MS patients, and how these genes/proteins can be modulated by current DMTs in MS.

Newly acquired molecular details of how hemostasis components trigger neuroinflammation and neurodegeneration could in turn favor the development of novel therapeutic approaches to ameliorate the disease evolution, favored by the wealth of powerful inhibitors or potentiators of hemostasis used for treatment and prophylaxis in prothrombotic and hemorrhagic disorders.

## Author Contributions

NZ and RZ contributed to the study concept and design, critical revision of the manuscript for important intellectual content, and study supervision. DJ and FB contributed to the study concept and design, analysis and interpretation, critical revision of the manuscript for important intellectual content, and study supervision.

### Conflict of Interest Statement

RZ has received speaker honoraria and consultant fees from Genzyme-Sanofi, Novartis, Claret Medical, Celgene, and EMD Serono. He has received research support from EMD Serono, Genzyme-Sanofi, Claret Medical, Protembis, QuintilesIMS, and Novartis. The remaining authors declare that the research was conducted in the absence of any commercial or financial relationships that could be construed as a potential conflict of interest.
